# The chemical composition and heavy metal content of sesame oil produced by different methods: A risk assessment study

**DOI:** 10.1002/fsn3.2245

**Published:** 2021-03-17

**Authors:** Somayeh Kheirati Rounizi, Fateme Akrami Mohajeri, Hamdollah Moshtaghi Broujeni, Fatemeh Pourramezani, Sara Jambarsang, Hossein Kiani, Elham Khalili Sadrabad

**Affiliations:** ^1^ Zoonotic Diseases Research Center Department of Food Hygiene and Safety School of Public Health Shahid Sadoughi University of Medical Sciences Yazd Iran; ^2^ Food Hygiene and Quality Control School of Veterinary Medicine Shahrekord University Shahrekord Iran; ^3^ Food Health Research Center Hormozgan University of Medical sciences Bandar Abbas Iran; ^4^ Research Center of Prevention and Epidemiology of Non‐Communicable Disease Department of Biostatistics and Epidemiology School of Public Health Shahid Sadoughi University of Medical Sciences Yazd Iran; ^5^ Bioprocessing and Biodetection Lab Department of Food science and Technology University of Tehran Karaj Iran; ^6^ Nutrition and Food Security Research Center Shahid Sadoughi University of Medical Sciences Yazd Iran

**Keywords:** chemical characteristics, heavy metal content, risk assessments, sesame oil production method

## Abstract

The oil was extracted from sesame seed with two extraction methods. Traditional (Ardeh oil) and industrial method (cold pressing method: virgin and refined sesame oil) oil extraction was studied to compare the quality and heavy metal content of extracted oils. The chemical properties (fatty acid composition, peroxide, anisidine, acid values, and TOTOX) and heavy metal contents were investigated. The Hazard Quotient (HQ) and Hazard Index (HI) of heavy metal intakes were calculated. The results demonstrated that the predominant fatty acid in oil samples was oleic, linoleic, palmitic, and stearic acids. It was indicated the peroxide, anisidine, acid values, and TOTOX of oil samples were as the order of Ardeh oil > virgin sesame oil > refined sesame oil. The reduction pattern of Pb > Zn >Cu > Cd >As was reported in sesame seed. Although the oil refining had been greatly reduced the Pb of oil sample, but it had yet been much higher than the permissible levels set by Codex Alimentarius. The HQ and HI of all heavy metals were less than one, but they were higher in Ardeh oil compared to others. It is necessary to monitor the presence of heavy metal contaminants and the quality of imported sesame seeds prior to oil preparation.

## Graphical abstract

In current manuscript, different processing methods of sesame oil in chemical characteristics and heavy metals have been studied.

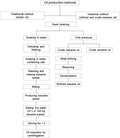

## 1 INTRODUCTION

Fats and oils are essential in human dietary which is considered as an important sources of energy, fat‐soluble vitamins, and essential fatty acids (Ghazani et al., 2013; Llorent‐Martínez et al., 2011). Sesame seed (*Sesamumindicum L*.), an important oil crops, has been cultivated in Asian countries to be used as cooking oil and seasoning ingredients(Lee et al., 2010). The anti‐inflammatory activity and anti‐cancer effects of sesame oil have been proved (Hashempour‐Baltork et al., 2017). Although sesame oil is rich in unsaturated fatty acids, it possesses high oxidative stability rather than other vegetable oils (Lee et al., 2010). The high oxidative stability of sesame seed is attributed to the presence of lignans such as sesamolin, sesamin, sesamol, and tocopherols (Lee et al., 2010). Although, the stability of sesame oil to oxidation and oil quality depends on several factors such as sesame seed growth condition (soil, environment, and genotype), seed treatments, oil extraction methods, processing condition, and presence of trace elements (Lamas et al., 2014; Llorent‐Martínez et al., 2011). Due to development of industries and increasing environmental pollution in last decades, the food contamination by heavy metals has become an important issue (Fakoor Janati et al., 2011). Along with contamination of oil seeds through soil, fertilizers, and geographical condition, oil extraction process and method of extraction could be effective in heavy metal content of extracted oil (De Leonardis et al., 2000; Pehlivan et al., 2008). The industrial method of oil extraction is cold pressing, which is squeezing of the seeds without applying heat. It was shown that refined oil has high quality and stability, free of any additives or chemical preservatives, and contains the most beneficial nutrients (Lamas et al., 2014; Martínez et al., 2017). In Iran, despite from the cold press method to extract the sesame oil, the traditional extraction method had been performed from ancient times. In the traditional method, the sesame hulls remove by soaking the sesame seed in solution of water and salt. The dehulled seeds would be roasted and grinded to produced Ardeh (Tahineh) which in combination with water and further squeezing produce the Ardeh oil (sesame paste oil) (Shahidi, 2005).

By considering that sesame seed could accumulate heavy metal (Hao et al., 2011), investigation about heavy metal content of its extracted oil and evaluation of possible risk assessments seem necessary. Therefore, in current study, heavy metals (lead, cadmium, zinc, arsenic, and copper) and fatty acids profiles of virgin, refined, and Ardeh oils as well as some other qualitative characteristics including acid, peroxide, anisidine, and TOTOX values were assessed. Consequently, the Hazard Quotient (HQ) and Hazard Index (HI) of individual and interactive effects of exposure to two or more contaminant, respectively, were evaluated.

## 2 MATERIALS AND METHODS

### 2.1 Sample preparation

#### 2.1.1 Preparation of Ardeh oil

**FIGURE 1:**
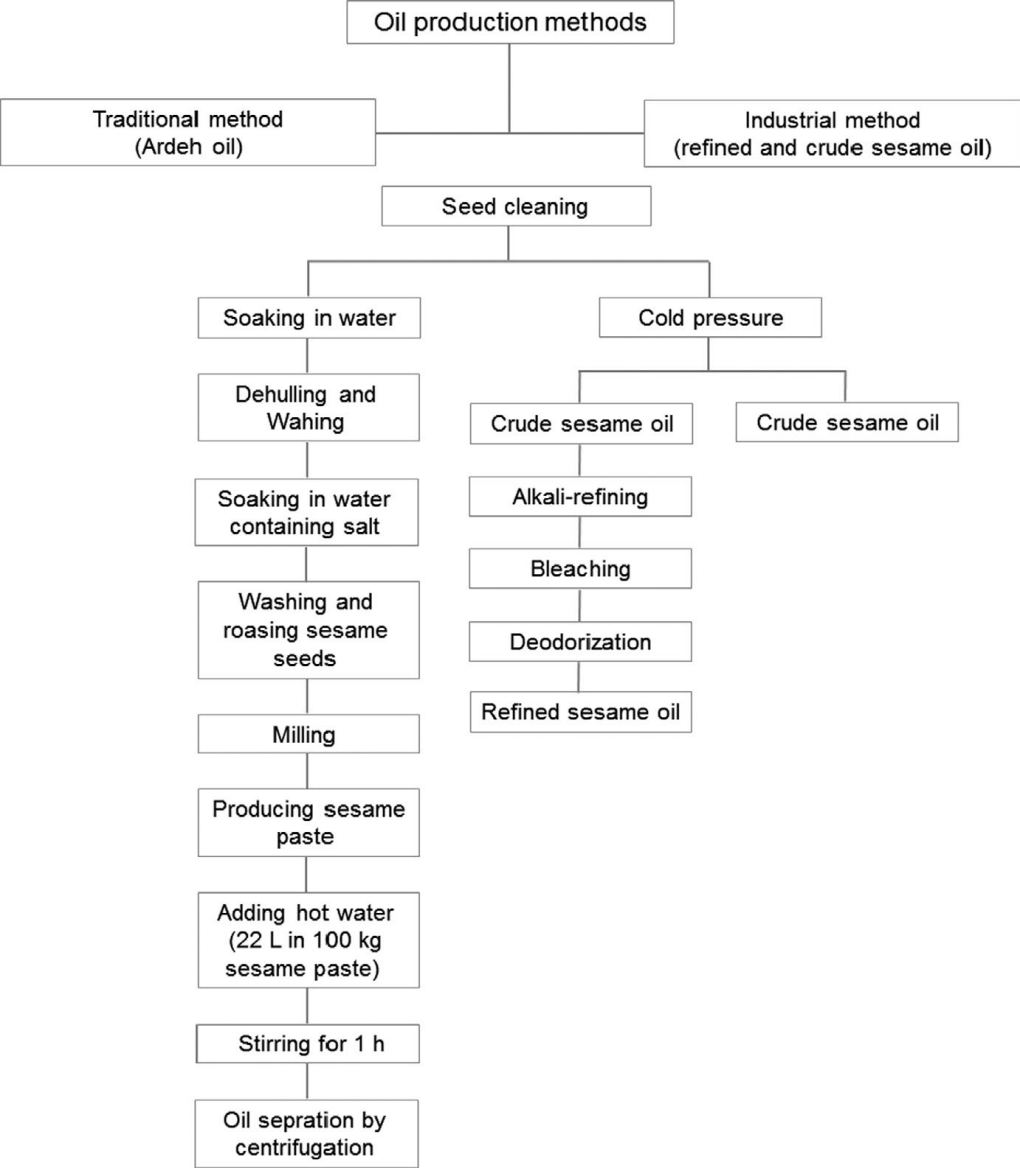
Scheme of the production of refined and crude sesame oils, and Ardeh oil by three methods

The oil samples were produced according to Figure 1. In order to prepare Ardeh oil (sesame paste oil), the sesame seeds were soaked in the mixture of water and salt to remove the hull. The dehulled sesame seeds were roasted at temperature less than 200°C, followed by grounding the sesame seed to produce sesame paste. The sesame paste was mixed with water in the ratio of 10:2.2, and the oil was separated by centrifuging (INSO, 2016).

#### 2.1.2 Preparation of virgin and refined sesame oil

The sesame seeds were put into a cold presser (temperature less than 40–45°C), and the oil was extracted under low pressure. Extracted oil was stored in storage tanks, and subsequently, oil filtration was conducted to isolate the waste. A sample of crude oil (virgin sesame oil) was prepared from sesame oil without further treatment. Then, the refined sesame oil was obtained after neutralization, bleaching, and deodorization (Botha et al., 2000).

### 2.2 Gas Chromatography–Flame Ionization Detection (GC‐FID)

The fatty acids composition of the samples was determined by Gas Chromatography–Flame Ionization Detection (GC‐FID, Yang Lin 6,500, and South Korea) and capillary column (120 m × 2.5 mm i.d.; 0.25 µm). Detector temperature was programmed from 90 to 240°C (seven minutes at 90°C; ten minutes at 150°C; 15 min at 200°C; and 20 min at 240°C), and held at 240°C for 50 min. Helium gas (99.99%) was used as the carrier at 20 ml/min, and hydrogen and air at a ratio of 1:30 were used as oxidant. The retention time of methyl ester of samples was compared with standard(Botha et al., 2000). The calculated oxidizability value (COX) was calculated according to percentage of unsaturated C18 fatty acids in oil by following equation (Fatemi & Hammond, 1980):

$$\text{COX value}=\left[1\times \left(C18:1\%\right)+10.3\times \left(C18:2\%\right)+21.6\times \left(C18:3\right)\right]/100.$$

### 2.3 Chemical properties

Acid value was measured according to AOAC standard (AOAC, 1999), peroxide value (PV) according to AOCS(AOCS, 1992), and Anisidine value (p‐AV) according to AOCS standard (AOCS, 2011). The TOTOX was evaluated according to the equation: TOTOX = 2PV + p‐AV.

### 2.4 Heavy metal analysis

#### 2.4.1 Reagent

All reagents were of analytical reagent grade. In order to decrease the risk of contamination, all glasswares were soaked in diluted nitric acid (10%) overnight and rinsed with distilled deionized water several times. In order to use the stock solution for calibration, multi‐elemental standard solution (1,000 mg/kg in nitric acid 0.5 N) was prepared.

#### 2.4.2 Microwave digestion

A total of 0.5 g homogeneous oil samples were digested with 5 ml of HNO_3_ (65%) and 2 ml H_2_O_2_ (30%) in microwave digestion system (15‐Sineo MDS, China) for 30 min according to following program in Table 1. The blank sample was prepared in the same procedure. The final volumes of sample were diluted to 10 ml by distilled deionized water (Zhu et al., 2011).

**TABLE 1:** Operating program for the microwave digestion of oil samples

Step	Temperature (°C)	Time (min)	Power (w)
1	130	10	400
2	150	5	400
3	180	5	400
4	200	10	400

#### 2.4.3 Heavy metal determination

The digested samples were injected to Inductively Coupled Plasma–Optical Emission Spectrophotometer (SPECTRO GENESIS model) to determine the heavy metal concentration. The accuracy of method was evaluated based on trace elements in standard reference material (NIST‐SRM 1577b bovine liver) (Uluozlu et al., 2009). According to achieved results, a good agreements were reported with certified values (Table 2). All analyses were done in triplicate (Khalili Sadrabad et al., 2018; Mohajer et al., 2020).

**TABLE 2:** Trace element concentrations in certified reference material (NIST‐SRM 1577b) and recovery of analyzed metals

Heavy metal (*n* = 3)	Certified value (µg/g)	Our value (µg/g)	Recovery (%)
As	0.05	0.047 ± 0.002	95.6
Pb	0.129	0.126 ± 0.007	97.67
Cd	0.5	0.45 ± 0.01	90.4
Zn	127	129 ± 9	101.5
Cu	160	158 ± 6	98.81

### 2.5 Health Risk Assessment

#### 2.5.1 Estimated daily intake (EDI) of metals

The EDI of metals was evaluated by considering the daily consumption of 25 g for oil according to World Health Organization (WHO). The body weight of adult was set to 70 kg in current study. The EDI was calculated according to following equation (Pourramezani et al., 2019; Zhu et al., 2011).

EDI = daily food consumption (g) ×concentration of metal (mg/kg)/ body weight (kg).

#### 2.5.2 Hazard Quotient (HQ)

The HI for non‐carcinogenic risk of metal can be calculated as following equation:

HQ=EDI/RfD

where according to FAO/WHO, the oral toxicity reference dose values (RfD) are 0.3, 1, 40, 300, and 3.75 µg/kg for arsenic, cadmium, copper, zinc, and lead, respectively (Harmanescu et al., 2011; Kamunda et al., 2016).

#### 2.5.3 Hazard Index (HI)

Exposure to two or more heavy metals may increase the interactive effects. The HI which shows the total non‐carcinogenic health risk of heavy metals was calculated as bellow (Pourramezani et al., 2019):

$$\text{HI}={\text{HQ}}_{1}+{\text{HQ}}_{2}+{\text{HQ}}_{3}+\ldots +{\text{HQ}}_{n}$$

### 2.6 Statistical analysis

The data were analyzed by the SPSS16 software. ANCOVA was used to investigate the effect of oil extracting methods.

## 3 RESULT AND DISCUSSION

### 3.1 Fatty acids composition

Due to technological and nutritional importance of fatty acid composition inedible oils, the fatty acid analyses are essential (Hashempour‐Baltork et al., 2017). It has been shown that several unsaturated fatty acids such as alpha‐linolenic are important in reduction of coronary heart disease incidence (Bucher et al., 2002). On the other hand, high levels of unsaturated fatty acids decrease the oxidation stability which is important indicator for controlling the quality of edible oils (Tan et al., 2002). In current study, the predominant fatty acids in three oils were linoleic acid, oleic acid, palmitic acid, and stearic acid (Table 3), which is consistent with the results of Yoshida et al (Yoshida et al., 2000) and Abou‐Gharbia (Abou‐Gharbia et al., 1997). It was shown that linoleic acid of Ardeh oil was significantly higher than virgin and refined sesame oil (*p* < .05). The oleic acid was evaluated significantly higher in virgin sesame oil with value of 41.35% (*p* < .05). Palmitic acid was predominant saturated fatty acid in refined sesame oil followed by virgin sesame oil. Margaric acid was only detected in Ardeh oil with value of 0.1%. In overall, it was reported that oil refining has slight effects on saturated and unsaturated fatty acids composition of oil which is in agreement with results in rubber seed (Achinewhu & Akpapunam, 1985), hazelnut oil (Karabulut et al., 2005), and groundnut oil (Aluyor et al., 2009). The results of Ji et al showed no differences in fatty acid profile of oils prepared from roasted and unroasted sesame seed (Ji et al., 2019) which is not in agreement with current study. The PUFA/SFA ratio shows the extent of polyunsaturated fatty acids and tendency to autoxidation (Méndez et al., 1996). Therefore, the lowest oxidative stability (high PUFA/SFA ratio) was evaluated in Ardeh oil with value of 2.43.

**TABLE 3:** Fatty acid composition of Ardeh, virgin sesame, and refined sesame oil

	Ardeh oil	Virgin sesame oil	Refined sesame oil
Palmitic acid	10.8 ± 0.001^a^	12.6 ± 0.2^b^	12.65 ± 0.25^b^
Palmitoleic acid	0.0 ± 0.0^a^	0.2 ± 0.001^b^	0.25 ± 0.05^b^
Margaric acid	0.1 ± 0.001^a^	0.0 ± 0.0^a^	0.0 ± 0.0^a^
Stearic acid	5.5 ± 0.001^a^	5.1 ± 0.2^a^	5.3 ± 0.1^a^
Oleic acid	40.4 ± 0.001^a^	41.35 ± 0.15^b^	40.3 ± 0.4^a^
Linoleic acid	41.8 ± 0.001^a^	40.45 ± 0.35^b^	40.4 ± 0.3^b^
α Linolenic acid	0.3 ± 0.001^a^	0.3 ± 0.001^a^	0.2 ± 0.001^a^
Arachidic acid	0.7 ± 0.001^a^	0.4 ± 0.001^a^	0.5 ± 0.001^a^
Behenic acid	0.2 ± 0.001^a^	0.1 ± 0.001^a^	0.1 ± 0.001^a^
SFA	17.3	18.2	18.55
MUFA	40.4	41.55	40.55
PUFA	42.1	40.75	40.6
PUFA/SFA	2.43	2.23	2.18
MUFA/PUFA	0.95	1.01	1.00
COX value	4.77	4.64	4.60

^a,b^ Different letters mean the significant differences in level of 5%.

According to result, the highest oxidation stability was shown in refined sesame oil seed which had the lowest COX value. The lowest oxidation stability belongs to Ardeh oil which can be attributed to high PUFA and low MUFA content of Ardeh oil. It was shown that oil extracted from whole sesame seed without dehulling could be stable in oxidation which is in agreement with results of Kamal‐Eldin (Kamal‐Eldin & Appelqvist, 1995) and Konsoula (Konsoula & Liakopoulou‐Kyriakides, 2010), although it was not in agreement with Tenyang results (Tenyang et al., 2017).

#### 3.1.1 Chemical properties of the oils

The chemical properties of the oils including peroxide value, FFA, Anisidine value, and TOTOX are shown in Table 4. According to the results, the highest and the lowest peroxide value were reported in Ardeh oil (2.17 meq/kg) and refined sesame oil (0.29 meq/kg), respectively. It was shown that all samples had a peroxide value within the permissible level which indicated relatively good oxidation stability. The high oxidation stability of oils could be attributed to presence of lignans (sesamol, sesamolin, and sesamin) and tocopherols (Gharby et al., 2017). The hull of sesame seed is rich in antioxidant compounds, and during oil extraction, antioxidants would transfer into oil and increase the oxidative stability (Abou‐Gharbia et al., 1997). Thus, the high peroxide value in Ardeh oil could be attributed to dehulling process of sesame seeds and heating during extraction. Therefore, the cold pressing and filtration process of oil would significantly reduce the peroxide value.

**TABLE 4:** Chemical properties of Ardeh, virgin sesame, and refined sesame oil

Chemical properties	Ardeh oil	Virgin sesame oil	Refined sesame oil
Peroxide value (meq O_2_/kg oil)	2.17 ± 0.26^a^	1.15 ± 0.22^ab^	0.29 ± 0.13^b^
Acid value (mg KOH/g oil)	4.18 ± 0.41^a^	3.68 ± 0.57^a^	0.067 ± 0.012^b^
Free fatty acid (% Oleic acid)	2.09 ± 0.2^a^	1.84 ± 0.31^a^	0.033 ± 0.01^b^
Anisidine value	1.84 ± 0.42^a^	0.84 ± 0.19^b^	0.6 ± 0.09^b^
TOTOX	6.18 ± 0.94^a^	3.14 ± 0.63^b^	1.18 ± 0.36^c^

Note

Different letters in each raw show significant differences at level of *p* <.05.

The acid value is introduced as an important method to determination of oil quality by measuring the free fatty acid content (Farhoosh et al., 2009; Tenyang et al., 2017). The acid value (AV) of refined and cold‐pressed virgin oils was set 0.6 and 4 mg KOH/g oil by Codex Alimentarius (Alimentarius, 2003). In the present study, Ardeh oil had the highest acid value compared with the two other oils, which can be attributed to the processing method. During Ardeh oil processing, the used water could increase the hydrolysis of triacylglycerols and releasing fatty acids. In addition, the high temperature during roasting the sesame seeds for Ardeh oil production could increase the rapid hydrolysis of triacylglycerols and accumulation of fatty acids (Suri et al., 2019). Therefore, during the Ardeh oil processing, large amounts of free fatty acids would be released which is consistent with achieved AV results in current study. The lowest acid value was reported in refined sesame oil which is in agreement with Aluyor et al (2009), Farhoosh et al (2009), and Suri et al (2019).

During the lipid oxidation, hydroperoxides rapidly decomposed to secondary metabolites which would be measured by p‐Anisidine value (p‐AV) (Tenyang et al., 2017). The p‐AV of Ardeh oil with 1.84 was significantly (*p* < .05) higher than other samples, which means the higher production of secondary oxidation metabolites in Ardeh oil. The refined sesame oil had the lowest p‐AV (0.6), which is due to the lesser PUFA concentration and lack of roasting stage during production. It was found that the p‐AV of oils contained high PUFA was much faster which is in agreement with Tenyang et al (Tenyang et al., 2017) and Yaacoub et al (Yaacoub et al., 2008). The TOTOX value shows the primary and secondary oil oxidation (Tenyang et al., 2017). The TOTOX value of Ardeh oil, virgin sesame oil, and refined sesame oil was determined 6.18, 3.14, and 1.18, respectively. The TOTOX value of refined sesame oil was significantly lower (*p* < .05) than other samples.

#### 3.1.2 Heavy metals content of oils

Heavy metals could enter the human body through the contamination in food chain. Presence of heavy metals in oils is related to oxidative deterioration and adverse effects on oil shelf life. The results of the analysis of heavy metals in sesame seed and different oil samples are presented in Table 5. The reduction pattern of Pb > Zn >Cu > Cd >As was reported in sesame seed. In overall, it was shown that the heavy metals in sesame seed were much higher than its extracted oils. The Cd and Zn content of virgin oil was estimated lower than other samples. In Ardeh oil, except for As and Zn, the higher amount of heavy metals was reported in comparison with two other oils. The reduction patterns of Pb and Cu from lowest to highest were found as refined sesame oil, virgin sesame oil, and Ardeh oil, respectively. Therefore, it was shown that the refining process could be considered as an efficient method in reduction of heavy metals which is in consistence with result of Akbari‐adergani et al (Akbari‐adergani et al., 2015) and Sedaghat et al (Sedaghat et al., 2018). The As content of all oil samples was in the permissible levels set by Codex Alimentarius (0.1 ppm in edible oils) (Alimentarius, 1999). The As content of sesame seed was estimated 0.042 mg/kg which is lower than results of Fakoor Janati research (Fakoor Janati et al., 2011). In the present study, the lowest As in Ardeh oil could be attributed to the process of soaking, rinsing, and peeling the sesame which caused in removing the As from its extracted oil. Different studies indicated the As reduction in soaked rice which is consistent with our results (Mihucz et al., 2007; Raab et al., 2009; Rahman et al., 2006).

**TABLE 5:** Heavy metal concentration (mg/kg) in sesame seed, Ardeh, virgin sesame, and refined sesame oil

Heavy metals (mg/kg)	sesame	Ardeh oil	Virgin sesame oil	Refined sesame oil
As	0.042 ± 0.02^a^	< 0.000698^b*^	0.016 ± 0.010^c^	0.016 ± 0.012^c^
Pb	3.27 ± 9.98^a^	0.37 ± 0.71^b^	0.25 ± 0.61^c^	0.20 ± 0.66^c^
Cd	0.058 ± 0.024^a^	0.021 ± 0.009^b^	0.0027 ± 0.0016^c^	0.0123 ± 0.0074^b^
Zn	19.9 ± 2.02^a^	< 0.00028^b*^	< 0.00028^b*^	0.085 ± 0.01^c^
Cu	3.47 ± 0.14^a^	0.145 ± 0.048^b^	0.144 ± 0.001^b^	0.136 ± 0.022^b^

Note

Wave length: Zn: 213.856; Pb: 261.418; Cd: 214.438, As: 228.812, Cu: 324.754 nm

The sign * showed that the results were estimated lower than Limit of Detection (LOD).

Different letters mean the significant differences in level of 5%.

However, the oil refining greatly reduced the amount of Pb of oil samples, but in all samples, it was much higher than the permissible levels set by Codex Alimentarius (0.1 ppm in edible oils). The Pb content of Ardeh, virgin sesame, and refined oils was 3.72, 2.59, and 2.33 ppm, respectively. It seems that the high amount of Pb in sesame seed was significantly removed during processing. According to Ashraf results, the As, Cd, Cu, and Zn concentration of sesame oil was reported 0.013, 2.36, 0.286, and 1.88 mg/kg, respectively (Ashraf & Khobar, 2014), which except for As, all heavy metals were higher than that of current study. In Angelova et al study, the amount of Pb, Cu, Zn, and Cd in sesame seed was reported in range of 0.5–1.9, 3.8–5.3, 20.4–26.2, and 0.04–0.1 mg/kg, respectively (Angelova et al., 2005). According to current research, except for Pb, other heavy metals were lower than results of Angelova research (Angelova et al., 2005). The concentration of Cu, Zn, Pb, and As of sesame oil samples in Zhu et al study was determined 0.028–0.039, 0.789–0.883, 0.014–0.018, and 0.015–0.019 mg/kg, respectively (Zhu et al., 2011). These differences in heavy metal content of sesame oils can be attributed to the genetic differences of sesame strains as well as agro‐climatic conditions (Mohammed et al., 2018). Therefore, due to the nature and characteristics of heavy metals and the ability to concentrate in different parts of plants, their transfer from sesame seed to its produced oil in various methods differs. In general, the different processing procedures such as soaking in water, roasting, dehulling of sesame seed, and refining could be considered as an effective way in reduction of heavy metal content of extracted oil.

#### 3.1.3 Determine health risk assessments

Intakes of heavy metal through food chain are related to heavy metal concentrations in food and amount of food consumed (Zhu et al., 2011). The possible health risk of sesame oil (virgin and refined) and Ardeh oil consumptions was evaluated. The Estimated Daily Intakes (EDI), Hazard Quotient (HQ), and Hazard Index (HI) of heavy metals in Ardeh oil, virgin sesame oil, and refined sesame oil are given in table 6. According to the results, the HQ values of Pb in oil samples were the highest value with the order of Ardeh oil > Virgin sesame oil > refined sesame oil. The HQ level of heavy metals in Ardeh oil, virgin sesame oil, and refined sesame oil showed the order Pb > Cd >Cu > As >Zn, Pb > As >Cu > Cd >Zn, and Pb > As >Cd > Cu >Zn, respectively. It was shown that the HI value of Ardeh oil (0.0448) was higher than other samples. The HQ and HI values related to all heavy metals in oils were less than one, which indicated no hazard in oil consumption which is in consistence with results of Sobhan Ardakani (Sobhan Ardakani, 2016) and Zhu et al (Zhu et al., 2011). Therefore, according to HQ and HI indexes, the consumption of studied oils is not harmful with regard to the presence of Cd, Pb, Cu, Zn, and As.

**TABLE 6:** Estimated Daily Intakes (EDI), Hazard Quotient (HQ), and Hazard Index (HI) of heavy metals in Ardeh oil, virgin sesame oil, and refined sesame oil from consumption of 25 g edible oils per day

Risk assessments	Heavy metal	Ardeh oil	Virgin sesame oil	Refined sesame oil
EDI	As	0.000249	0.0057	0.0059
	Pb	0.13	0.089	0.071
	Cd	0.0075	0.00096	0.0043
	Zn	0.0001	0.0001	0.030
	Cu	0.051	0.051	0.048
HQ	As	0.00083	0.019	0.0196
	Pb	0.0352	0.023	0.019
	Cd	0.0075	0.00096	0.0043
	Zn	0.00000033	0.00000033	0.0001
	Cu	0.001275	0.001275	0.0012
HI	∑HQ	0.0448	0.0442	0.0442

## 4 CONCLUSION

The linoleic acid, oleic acid, palmitic acid, and stearic acid were determined as the predominant fatty acids in three oils. According to the results, the highest peroxide, anisidine, and acid values were reported in Ardeh oil which is related to the processing method. The oil processing was introduced as an effective way in reduction of heavy metals. Based on the current results, the Pb concentration of all samples in all the studied oils was higher than the permissible limit. Although according to the health risk assessment, none of the samples were identified harmful to consumers; it is required to monitor the presence of heavy metal contaminants and the quality of imported sesame seeds prior to oil preparation. In overall, despite the Iranian public belief about the better quality of Ardeh oil in comparison to sesame oil, the results showed that Ardeh oil had the lowest quality in terms of chemical characteristics.

## CONFLICT OF INTEREST

There is no conflict of interest.
